# Noninvasive prenatal prediction of fetal haplotype with Spearman rank correlation analysis model

**DOI:** 10.1002/mgg3.1988

**Published:** 2022-05-29

**Authors:** Du Hanxiao, Sun Luming, Chen Songchang, Yang Jingmin, Zhang Yueping, Zhang Shuo, Chen Hongyan, Jiang Ning, Lu Daru

**Affiliations:** ^1^ State Key Laboratory of Genetic Engineering, School of Life Sciences Fudan University Shanghai China; ^2^ Department of Fetal Medicine & Prenatal Diagnosis Center Shanghai First Maternity and Infant Hospital, Tongji University School of Medicine Shanghai China; ^3^ Obstetrics and Gynecology Hospital, Fudan University Shanghai China; ^4^ Key Laboratory of Birth Defects and Reproductive Health of National Health and NHC Key Laboratory of Birth Defects and Reproductive Health, Chongqing Population and Family Planning Science and Technology Research Institute Chongqing China; ^5^ Shanghai WeHealth BioMedical Technology Co., Ltd. Shanghai China

**Keywords:** haplotype, multiple‐PCR, Spearman rank correlation analysis model, targeted hybrid capture

## Abstract

**Background:**

Noninvasive prenatal testing (NIPT) has been widely used clinically to detect fetal chromosomal aneuploidy with high accuracy rates, gradually replacing traditional serological screening. However, the application of NIPT for monogenic diseases is still in an immature stage of exploration. The detection of mutations in peripheral blood of pregnant women requires precise qualitative and quantitative techniques, which limits its application. The bioinformatic strategies based on the SNP (single nucleotide polymorphism) linkage analysis are more practical, which can be divided into two types depending on whether proband information is needed. Hidden Markov Mode (HMM) and Sequential probability ratio test (SPRT) are suitable for families with probands. In contrast, methods based on databases and population demographic information are suitable for families without probands.

**Methods:**

In this study, we proposed a Spearman rank correlation analysis method to infer the fetal haplotypes based on core family information. Allele frequencies of SNPs that were used to construct parental haplotypes were calculated as sets of nonparametric variables, in contrast to their theoretical values represented by a fetal fraction (FF). The effects on the calculation of the fetal concentration of two DNA enrichment methods, multiple‐PCR amplification, and targeted hybrid capture, were compared, and the heterozygosity distribution of SNPs within pedigrees was analyzed to reveal the best conditions for the model application.

**Results:**

Predictions of the paternal haplotype inheritance were in line with expectations for both DNA library construction methods, while for maternal haplotype inheritance prediction, the rates were 96.55% for method multiple‐PCR amplification and 95.8% for method targeted hybrid capture.

**Conclusion:**

Positive prediction rates showed that the maternal haplotype prediction was not as accurate as paternal one, due to the large amount of maternal noise in the mother's peripheral blood. Although this model is relatively immature, it provides a new perspective for noninvasive prenatal clinical tests of monogenic diseases.

## Graphical abstract

In this study, we proposed a Spearman rank correlation analysis method to infer the fetal haplotypes based on core family information. Predictions of the paternal haplotype inheritance were in line with expectations. Although this model is relatively immature, it provides a new perspective for non‐invasive prenatal clinical tests of monogenic diseases.

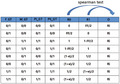

## 1 BACKGROUND

Prenatal diagnosis refers to the detection of the developmental status of the embryo before the fetus is born. For treatable diseases, with this diagnosis, we can choose an appropriate time for intrauterine treatment; for non‐treatable diseases, an informed choice can be made. In traditional prenatal diagnosis, fetal samples are obtained through invasive procedures, such as amniocentesis or chorionic sampling, with long detection cycles and risks of miscarriage, bringing a certain psychological burden to the pregnant women and their families. The discovery of fetal‐derived cell‐free DNA fragments in maternal peripheral blood provides new ideas for noninvasive prenatal testing (NIPT). Combined with biological information analysis, NIPT extracts total cell‐free DNA (cfDNA) from the peripheral blood of pregnant women, and identifies the fetus' cfDNA (cffDNA) to determine whether it has chromosomal ploidy changes or carries one or more specific disease‐causing genes.

Lo et al. (1997) detected the Y chromosome‐specific *SRY* gene in the peripheral blood of a pregnant woman carrying a male fetus through fluorescence quantitative PCR technology (Lo et al., 1997), which confirmed the presence of a small amount of cffDNA in the maternal peripheral blood for the first time. They are believed to come from placental trophoblast cells or naturally shed fetal cells, which can be detected at 7 weeks of pregnancy. The concentration of cffDNA increases with the increase in gestational age with a short half‐life period of 2 h. Compared to DNA fragments of maternal origin in plasma, which are about 166 bp, the DNA fragments of fetal origin are shorter, concentrated at 143 bp. Moreover, DNA fragments derived from mothers and fetuses tend to break at different chromosomal position coordinates (Chan et al., 2016), which could be used as the basis for distinguishing between two different sources of cell‐free DNA (cfDNA).

At present, NIPT has been widely used clinically, reducing the risk of miscarriage caused by invasive prenatal diagnosis. The detection rate of traditional serological screening is 60%~70%, with a low detection rate and a high false‐positive rate. The accuracy rates of NIPT for trisomy 21, 18, and 13 were 99.4%, 97.4%, and 92.5% with a false‐positive rate of 0.2%, 0.5%, and 0.8%, respectively (Health Quality Ontario, 2019; Sun et al., 2019; Vrachnis et al., 2014). Due to its high accuracy and low false‐positive rate, NIPT has been widely used in clinical prenatal screening and is gradually replacing serological screening. However, NIPT is still not a substitute for invasive prenatal diagnosis techniques, and serological screening remains a standard for clinical prenatal testing.

On top of being used for aneuploidy testing etc., NIPT can be applied for monogenic diseases. There are generally two strategies: one is to detect the mutation site of the disease‐causing gene directly; the other is to detect multiple SNPs (single nucleotide polymorphisms) linked to the disease‐causing site to determine whether the fetus has inherited the chromosome fragments with pathogenic mutations.

Lun et al. (2008) proposed the theory of the relative mutation dose method (RMD) (Lun et al., 2008) for the first time. RMD detects pathogenic mutation sites quantitatively using digital PCR or next‐generation sequencing (NGS). Based on the analysis of balance or imbalance of wild‐type and mutant alleles in maternal plasma, RMD enables the detection of, for example, β‐thalassemia (Xiong et al., 2018) and sickle cell anemia (Barrett et al., 2012). Through a unique barcode adapters and reverse primers design, and a unique counting method, Lv et al. (2015) developed a circularized single‐molecule amplification and re‐sequencing technology (cSMART). This technique is used to achieve the qualitative and absolute quantification of one single molecule in the mutation region. It has been applied to prenatal testing of phenylketonuria (Duan et al., 2019), non‐syndromic deafness (Han et al., 2017), and other single‐gene genetic diseases.

Instead of measuring the allelic alterations related only to one specific mutation site, Lam et al. (Lam et al., 2012) established a method for relative haplotype analysis (RHDO). RHDO is used to construct haplotypes through adjacent SNP sites on the same chromosome and conduct quantitative analysis using a second‐generation sequencing platform (NGS). Compared with the RMD approach, RHDO analyzes a range of SNP allele counts within a haplotype, which undoubtedly makes the statistical test more robust. This strategy was successfully applied in several types of monogenic diseases, such as spinal muscular atrophy (Parks et al., 2017), Duchenne and Becker muscular dystrophies (Parks et al., 2016), and Hunter syndrome (Hui et al., 2017).

RHDO requires information about the core family to construct the parental haplotypes. However, in families without a proband, additional methods are needed to construct the haplotype of both parents. Recently, long‐read sequencing methods have emerged (Ameur et al., 2019; van Dijk et al., 2018). The Oxford Nanopore sequencing technology (Jiang et al., 2021; Kuderna et al., 2019) enables the sequencing of regions that spans multiple heterozygous SNPs, making the parental haplotype analysis more accurate, and less complex. Currently, we have several options to construct haplotypes based on databases, such as the 1000G CHS haplotype dataset and SHAPEIT2 (Li et al., 2019), on population datasets (Chen et al., 2021), or the characteristics of the DNA fragments (Rabinowitz et al., 2019).

In this study, we selected β‐thalassemia as the focused disease and established a Spearman rank correlation analysis method to infer the two fetal haplotypes inherited from the father and mother separately with the additional information from probands. We compared the effect on the fetal concentration calculation of two DNA enrichment methods, multiple‐PCR amplification, and targeted hybrid capture, and analyzed the heterozygosity distribution of SNPs within pedigrees to reveal the best conditions for the model application.

## 2 MATERIALS AND METHODS

### 2.1 General workflow

Multiple‐PCR amplification strategy and targeted hybrid capture method were used for DNA enrichment for parental genomic DNA, proband's genomic DNA, and maternal cfDNA. Parental genomic DNA sequencing information was used to separately construct the parental haplotypes with the additional information from the proband. Maternal cfDNA sequencing results were applied to predict fetal genotypes. Haplotypes of the amniotic fluid sample were used to validate the results of the prediction model.

### 2.2 Sample collection

We recruited 130 couples during mid‐pregnancy after appropriate counseling and provided written informed consent. Peripheral blood of 10 ml were collected from each pregnant woman, and 2 ml of peripheral blood or 2 ml of saliva were collected from each of their husbands. To validate the result of deduction, 5 ml of amniotic fluid were collected from each pregnant woman during the prenatal examination.

### 2.3 DNA extraction

Peripheral blood of 5 ml from pregnant women were collected. The anticoagulated blood was divided into three layers (lower layer of red blood cells, the intermediate white blood cells, and the upper layer of plasma). The upper plasma was transferred for secondary centrifugation with the same parameters. The liquid supernatant was transferred and labeled for subsequent extraction of maternal cfDNA. The intermediate white blood cells were transferred into a clean 2 ml Eppendorf tube (EP tube) and labeled for subsequent extraction of genomic DNA of the mother.

The cfDNA was extracted with DK607 cell‐free DNA extraction kit (Lifefeng, Shanghai, China); Parental genomic DNA was extracted with DK601 blood genomic DNA mini extraction kit (Lifefeng, Shanghai, China); Fetal DNA was extracted from amniotic fluid with DK803 trace DNA extraction kit (Lifefeng, Shanghai, China). All experimental operations were conducted following the manufacturer's instructions with the default parameters.

### 2.4 Panel design

In this study, β‐thalassemia was selected as the focused disease and *HBB* (OMIM accession number: 141900; GenBank: U01317.1[62,137‐63,742]) was selected as the target gene for the validation of the methodology. The *HBB* gene is located between chr11:5,246,694 and chr11:5,248,301, with a total length of 1607 nucleotides, and contains three exons.

In multiple‐PCR panel design, SNPs inside *HBB* and in the range of 200 KB of the upstream and downstream of the target gene were selected and filtered according to the following criteria, (a) minor allele frequency (MAF) ranged from 0.3 to 0.7 in both 1000 Genome Database and Gnomad Database; (b) the GC content of the target area was between 0.4 and 0.55; (c) areas where DNA fragments were duplicated were avoided; (d) the occurrence of five consecutive identical bases in the enrichment segments was avoided; (e) adjacent SNP loci were separated by at least 10 KB; (f) their linkage disequilibrium parameter was greater than 0.8 verified by the Haploview. To increase the diversity of SNPs in the panel, 300 SNPs of other chromosomes on the genome were selected and filtered with the same criteria. Specific primers were designed, synthesized, and homogeneity adjusted for the screened areas by Moregene Biotechnology Co., Ltd, Shanghai, China.

In the targeted hybrid capture panel design, the full length of the *HBB* gene was bound by the probes with 1X coverage designed and synthesized by Twist Bioscience Corp. In the 100 KB range of the upstream and downstream of *HBB*, SNPs were selected with the same criteria as in multiple‐PCR panel design and covered by 1X probes.

### 2.5 DNA library construction and next‐generation sequencing

Quantification of genomic DNA was completed according to the manufacturer's protocol. Fragmented DNA of 200 ng or cfDNA were used as inputs for the library preparation. In the multiple‐PCR amplification process, the average length of the amplified template DNA fragments was approximately 120 bp after two rounds of PCR amplification. The first round was to enrich the target DNA fragments from whole‐genome DNA mixtures, and the second round was to add sequencing adaptors and sample‐specific barcodes to both ends of DNA fragments. After quality control and purification, the final PCR product was quantified by a Qubit® 3.0 Fluorometer (Life Technologies). In the target hybrid capture process, the genomic DNA was fragmented, with their ends repaired. After adding dA and adaptors to their ends, the small fragments were purified and amplified. Then the target area was enriched, eluted, and purified with Twist Hybridization and Wash Kit and Reagents.

The amplified and captured libraries were both sequenced on NextSeq 500 (Illumina) according to the manufacturer's recommendations using a pair‐end 150 bp protocol. The DNA library construction and the NGS were completed by We‐Health Biopharmaceutical Technology Co., Ltd, Shanghai, China.

### 2.6 Analysis of raw sequencing data

Parental genomic DNA and prenatal cfDNA were sequenced separately. Quality control of sequenced libraries was implemented through FastQC (version 0.10.1). The raw data were first aligned to the human reference genome (hg19, NCBI37) using the BWA software (version 0.7.17) with default parameters. SAMtools (version 1.9) was used to convert, sort, and index the alignment “bam” files. Steps of duplicates marking and removing were ignored due to the targeted DNA enrichment method of multiple‐PCR amplification. SNPs calling was then performed using SAMtools mpileup with parameters of mapping quality and base quality larger than 20.

### 2.7 Estimation of the cffDNA fraction

Accurate assessment of the concentration of the fetal‐derived DNA was essential to the detection of fetal haplotype. Based on their alleles depths or read counts, SNPs within the enrichment region that were homozygous for different alleles in each parent were used to calculate the fetal fraction (FF), such as 0/0 of mother genotype and 1/1 of father genotype. As for each SNP site, read counts of two alleles were marked as *p* and *q*. Specifically, *p* refers to read counts of the allele with the maternal genotype and *q* refers to read counts of the allele with the paternal genotype. According to the maximum likelihood estimate method, the fetal fraction could be calculated by formula $\mathrm{FF}=2\sum q/\sum \left(p+q\right)$.

### 2.8 Paternal haplotype construction

Following Mendelian inheritance, parental haplotype could be acquired through the genotype information of the parents and their first child (New et al., 2014). Paternity tests were carried out before inferring the fetal haplotype. Maternal and paternal haplotypes were drawn separately. SNPs within the enrichment region that were heterozygous in mother and homozygous in father were used to construct the maternal haplotype, such as AB of mother genotype and AA or BB of father genotype. In contrast, SNPs that were heterozygous in the father and homozygous in the mother were used to obtain the paternal haplotype. Combined with the genotype of the proband, haplotypes of each parent that were transmitted to their firstborn were distinguished from the others not inherited. Haplotypes that were inherited and not inherited by the proband were called H0 and H1, accordingly (Yang et al., 2020).

### 2.9 Application of Spearman rank correlation analysis model in fetus haplotype inference

In statistical language, Spearman rank correlation coefficient, *ρ*, refers to a nonparametric index that measures the dependence of two variables. It evaluates the correlation of two statistical variables using a monotone equation. Compared with the strict assumptions such as normal distribution, linear constraint, and homoscedasticity required by the Pearson correlation coefficient, the Spearman correlation coefficient does not require strict parametric assumptions, which leads to a wider applicability. The basic equation is:

$$\rho =\frac{\sum_{i}\left(x_{i}-\bar{x}\right)\left(y_{i}-\bar{y}\right)}{\sqrt{\sum_{i}{\left(x_{i}-\bar{x}\right)}^{2}\sum_{i}{\left(y_{i}-\bar{y}\right)}^{2}}}$$

where *ρ* represents the Spearman rank correlation coefficient; $\bar{x}$ and $\bar{y}$ refers to the average of the two sets of elements; *x*_*i*_ and *y*_*i*_ represent the ranks of the *i* element in datasets *x* and *y*, respectively.

In this study, allele frequencies of haplotype informative SNPs were calculated as sets of nonparametric variable, in contrast to their theoretical values represented by FF, according to their alleles' depths or read counts. As for each SNP site, read counts of two alleles were marked as *p* and *q*. Specifically, *p* refers to read counts of the allele with the reference genotype of hg19 and *q* refers to read counts of the allele with the alternative genotype of hg19. According to the maximum likelihood estimate method, the FF could be calculated by $R=\sum q/\sum \left(p+q\right)$.

**FIGURE 1:**
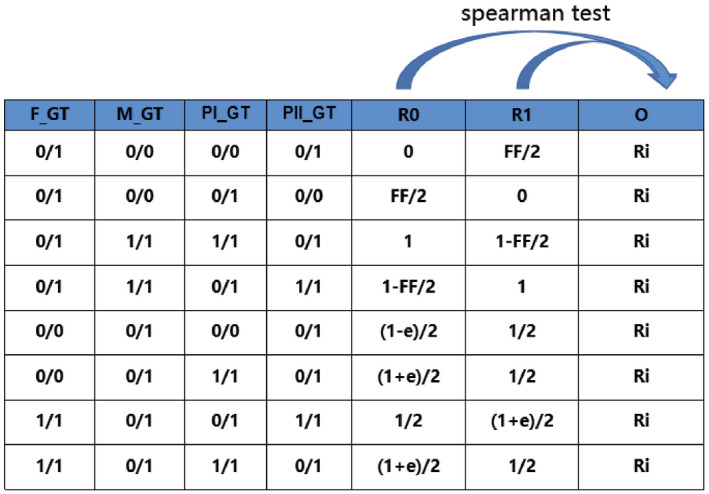
Schematic diagram of the inference of parental haplotype inheritance by Spearman's rank correlation analysis model.

Taking the inheritance of paternal haplotype as an example, in the inheritance of paternal haplotype, assuming that fetal haplotypes were H0 and H1, the expected frequencies of mutagenic alleles in maternal peripheral blood cfDNA sequencing were expressed as a function of FF, and classified as E0 and E1, respectively. Thus, the mutation allele frequency of these loci obtained from maternal peripheral blood cfDNA raw sequencing data was calculated and marked as O. In allusion to these two inheritances of haplotype, the Spearman rank‐sum test was performed between the observed values (O) and the two expected values (E1 and E2). The most suitable haplotype was determined based on the result of these two expected values using appropriate criteria. Deduction of the inheritance of maternal haplotype was performed by the same principle as paternal haplotype inheritance deduction (Figure 1).

## 3 RESULTS

### 3.1 Sample characteristics

We recruited 130 mid‐pregnancy couples after appropriate counseling and provided written informed consent. The minimum age of recruited pregnant women was 23 years old, the maximum was 43 years old, the average age was 32 years old; The gestational age of pregnant women recruited ranged from 11 to 24 weeks, with an average of 18 weeks. Karyotype tests of all amniotic fluid were normal.

Multiple‐PCR amplification of DNA enrichment strategy was applied to 95 of 130 families who had their prenatal care at Shanghai First Maternity and Infant Hospital; targeted hybrid capture of DNA enrichment strategy was applied to 25 of 130 families, who had their prenatal care at the International Peace Maternity & Child Health Hospital of China welfare institute. The DNA libraries quantification of these three groups is shown in Table 1.

**TABLE 1:** Quantification of DNA library acquired by multiplex PCR amplification and target hybrid capture

Sample	Mean ± SD (ng/ul)	Mean ± SD (ng/ul)
Father	22.49 ± 8.78	13.51 ± 2.87
Mother	23.51 ± 9.32	11.41 ± 3.15
Fetal	9.85 ± 3.44	21.45 ± 6.2
Plasma	17.88 ± 6.93	8.33 ± 5.76

### 3.2 Sequencing data characteristics

Sequencing depth varied depending on the DNA enrichment strategies (Tables 2 and 3). Data size represents the amount of raw data produced by the sequencer with units of MB. Average depth represents the average coverage depth of reads in the target areas. The mapping rate indicates the ratio of reads mapping the target areas to the total number of reads. ≥1X, ≥100X, and ≥500X indicate the ratio of areas with depths of ≥1X, ≥100X, and ≥5000, respectively to the total target areas.

**TABLE 2:** Statistics of raw sequencing data acquired by multiplex PCR amplification^a^

Sample	Data size (MB)	Average depth (X)	Mapping rate (%)	≥1X (%)	≥100X (%)	≥500X (%)
Father	79.73	4133.24	53.56	98.78	96.53	30.99
Mother	83.45	4349.71	53.65	98.81	96.50	33.47
Fetal	75.83	3756.50	52.55	98.83	97.53	25.67
Plasma	475.60	16,816.98	51.19	99.08	98.14	62.78

^a^ The full sequence of *HBB* (GenBank: U01317.1[62,137‐63,742]) and 200 KB of its upstream and downstream were selected as reference sequence.

**TABLE 3:** Statistics of raw sequencing data acquired by target hybrid capture^a^

Sample	Data size (MB)	Average depth (X)	on_target (%)	≥1X (%)	≥100X (%)	≥500X (%)
Father	373.95	399.32	26.47	99.20	88.81	0.87
Mother	459.59	481.02	26.11	99.33	90.79	1.98
Fetal	457.54	356.73	26.74	99.42	87.07	1.66
Plasma	1412.13	392.66	18.74	99.69	83.72	3.87

^a^ The full sequence of *HBB* (GenBank: U01317.1[62,137‐63,742]) and 100 KB of its upstream and downstream were selected as reference sequence.

The proportion of cffDNA was relatively small due to a large amount of maternal cfDNA (noise in the plasma sample), therefore plasma samples were sequenced with a deeper coverage. All the samples showed the same pattern of DNA amplification with the same DNA enrichment strategy, depending on the design of the primers or probes and the content of GC in the targeted region.

### 3.3 Calculation of fetal concentration

**FIGURE 2:**
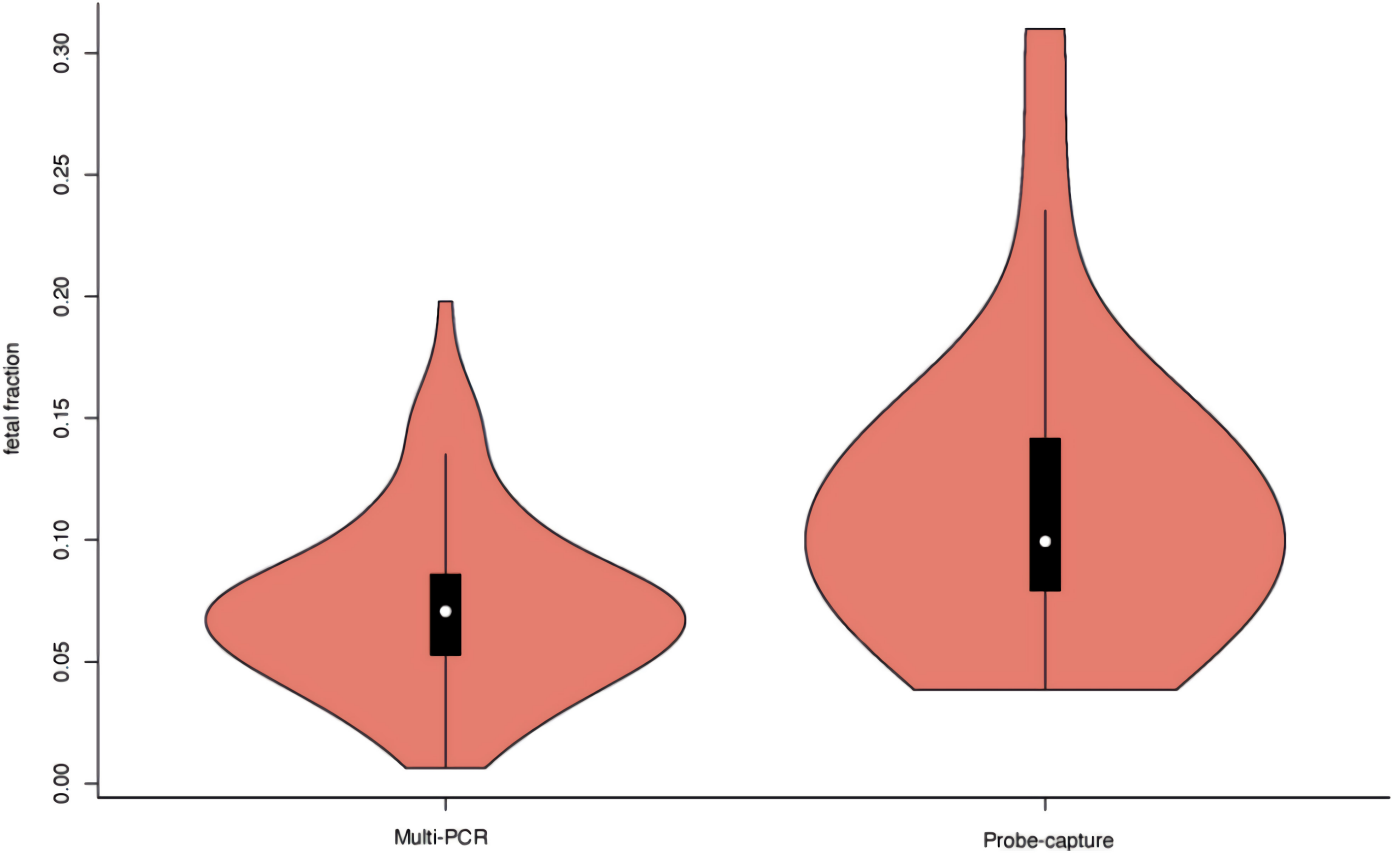
Comparison of fetal fraction calculated by multiplex PCR amplification and probe hybrid capture.

Mean concentrations of cffDNA in maternal plasma and their standard deviations were 7.32% ± 0.03, and 11.56% ± 0.06 for families with DNA enriched by multiple‐PCR amplification and by targeted hybrid capture, respectively (Figure 2). The former showed a lower trend compared with the latter or compared to clinical experience, presumably because the template cfDNA strand was too short to bind to pair of primers simultaneously, leading to its loss or an amplification failure.

### 3.4 Informative SNP statistics and parental haplotypes construction

Paternity tests were carried out before inferring the fetal haplotype. Here, we followed Mendelian genetics, where offspring receive dominant or recessive parental alleles. When analyzing the family with ID HBB‐722, we found that the number of sites with incorrect information was up to 30, counting 33.3% of total informative loci, thus this family was excluded from the follow‐up analysis.

**FIGURE 3:**
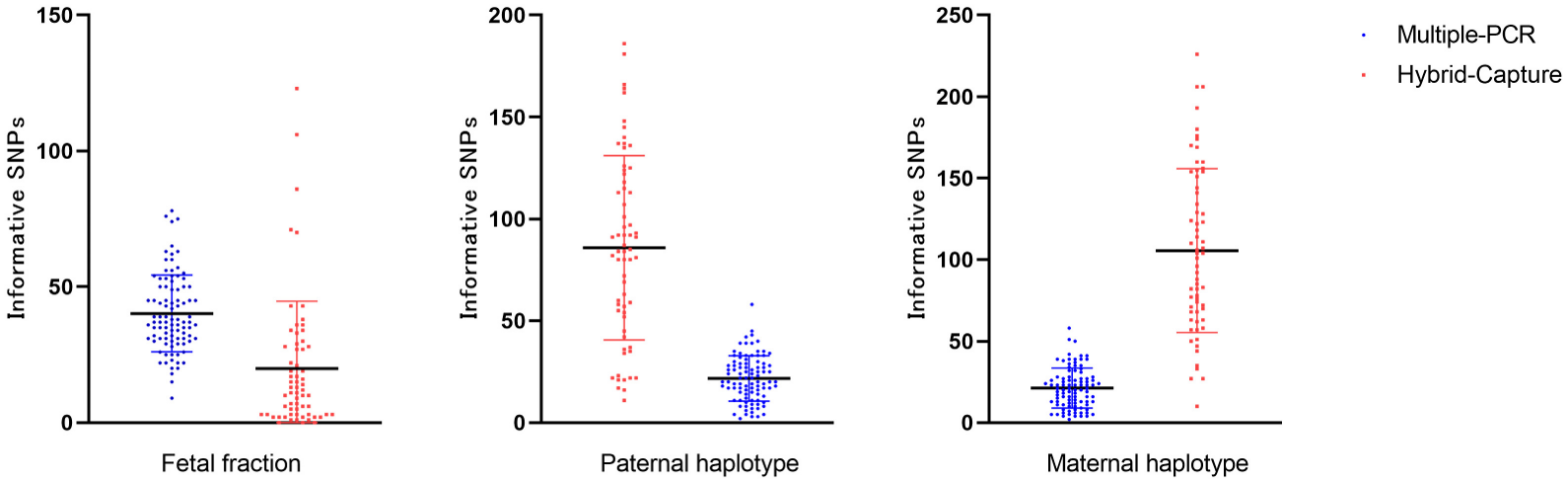
Distribution of numbers of informative SNPs of two different DNA enrichment strategies.

Lengths of DNA fragments enriched had different distributions due to the different principles of the two DNA enrichment strategies applied, leading to several SNPs with effective information. The distribution of informative SNPs of the two different DNA enrichment strategies is shown in Figure 3.

### 3.5 Deduction of fetal haplotypes

Pedigrees with informative SNPs of more than three were selected to construct parental haplotypes, and pedigrees that did not satisfy this requirement were discarded. Combined with the genotype of the proband, two independent haplotypes of the father or the mother were constructed separately.

During the sample collection process, the sample of the proband was replaced by the amniotic fluid after karyotype verification. It presented no chromosomal recombination in the targeted area. Due to the lack of proband in some families, the expected parental haplotype of the fetal inheritance should both be H0. While the expected haplotype of the fetus was based on the genotype of the proband in complete families, haplotypes that were the same and not the same with the haplotype of the proband were called H0 and H1, respectively.

As the concentration of the cffDNA in the maternal peripheral blood increased, the separation ratio of the two alleles at one SNP locus also increased. When the fetal concentration was less than 3%, it was impossible to accurately distinguish between the cfDNA derived from the mother and the fetus in the peripheral blood of the pregnant woman. Similarly, when the number of effective SNP sites was too small, the test result was not statistically significant. Therefore, the number of effective SNP sites greater than three was selected as a standard.

**FIGURE 4:**
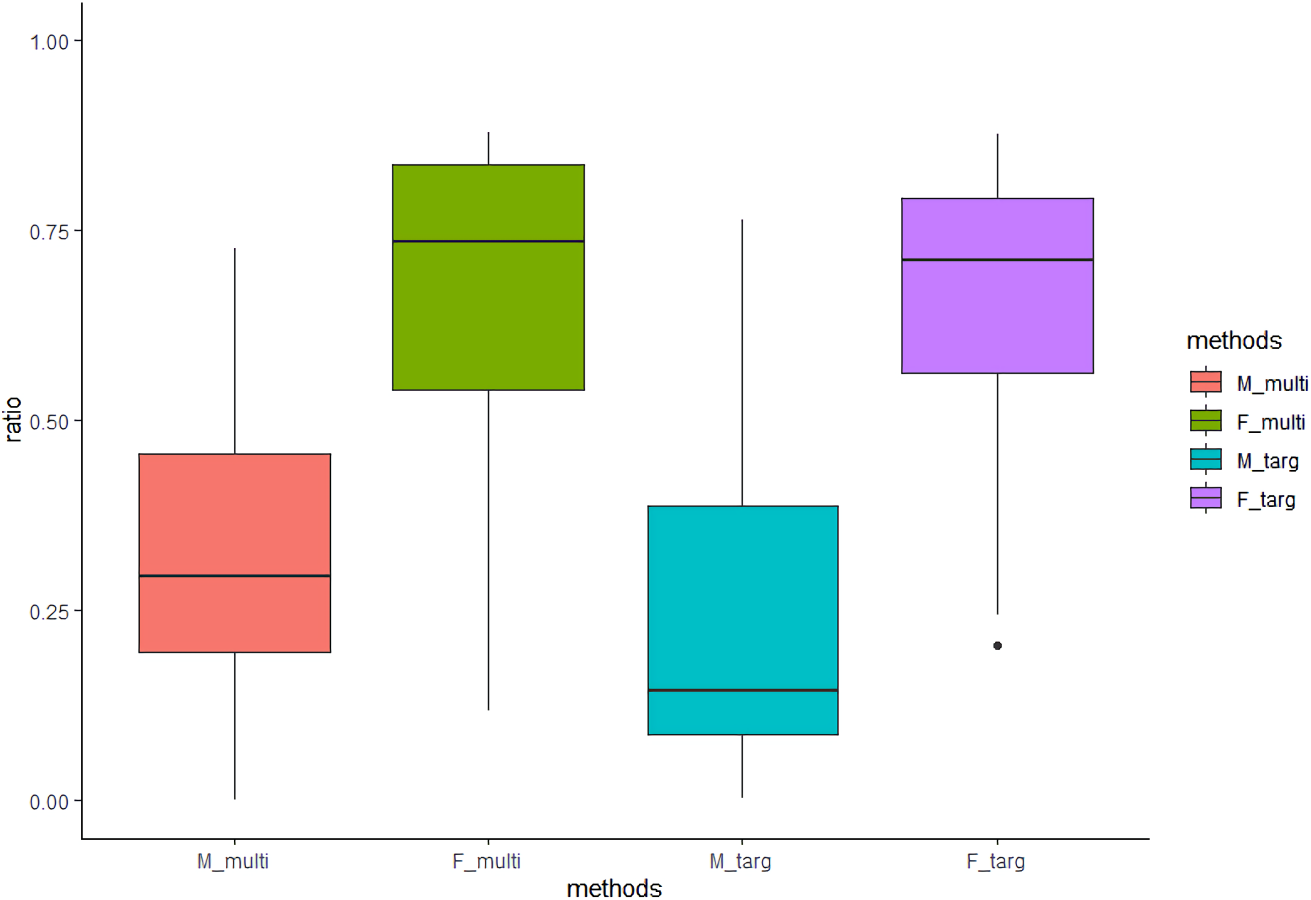
Statistics of the spearman rank correlation coefficient *ρ* of the parental haplotype inheritance by different methods.

Spearman rank correlation analysis model demonstrated that when inferring the haplotype inherited from the father, there was a positive prediction rate of 100% for both DNA enriched methods. In contrast, when inferring the haplotype inherited from the mother, there was a positive prediction rate of 96.55% in families whose DNA was enriched by multiple‐PCR amplification, and 95.8% enriched by targeted hybrid capture (Table 4). The distribution of the spearman rank correlation coefficient *ρ* of each pedigree is shown in Figure 4. The abscissa represents the parental haplotype inheritance by different methods, the ordinate represents the Spearman rank correlation coefficient, which ranges from 0 to 1.

**TABLE 4:** Statistics of fetal haplotype inheritance of each paternal haplotype achieved by multiplex PCR amplification and target hybrid capture

	Multiplex PCR amplification		Probe hybrid capture	
	Paternal haplotype	Maternal haplotype	Paternal haplotype	Maternal haplotype
Pedigrees recruited	94	94	24	24
FF <3%	6	6	0	0
Number of Informative SNPs <3	1	1	0	0
Fail	0	3	0	1
Success	87	84	24	23
Positive prediction rate	100%	96.55%	100%	95.8%

## 4 DISCUSSION

The fetal concentration calculated using the multiple‐PCR amplification library construction method was smaller compared to the targeted hybrid capture library construction method. This may be due to the lack of information about the break site of the original DNA fragments in the amplified product when enriching the target DNA with multiple‐PCR amplification. SNPs within the enrichment region that were homozygous for different alleles in each parent were used to calculate the FF. Combined with the distribution of SNPs in the population, the frequency of alleles with the genotype of 1/1 was lower than that of 0/0 or 0/1. This may result in a smaller number of SNP sites used to calculate the FF.

Moreover, the length of cfDNA in the peripheral blood of pregnant women is mostly concentrated at 166 bp, among which, cffDNA fragments were mostly concentrated at about 140 bp. When performing DNA amplification and enrichment, there may be cases where small fragments of free DNA template strands were too short to combine with two primers at the same time. If only one end of the primer is bound to the small fragment of cfDNA, the fragment fails to amplify, as a result, the concentration of cffDNA in the peripheral blood of pregnant women is underestimated.

When the genotype is heterozygous, the separation ratio of alleles was significantly smaller in pedigrees using the multiple‐PCR amplification library construction method compared to the targeted hybrid capture library construction method. It is speculated that when designing a multiple‐PCR amplification panel, the selection of primer sites is generally upstream and downstream of the SNP site, and the amplified fragment is about 70 bp. When designing a panel for probe hybridization capture, the coverage of the probes was generally 1X. As a result, alleles complementary to the probes sequence should have been preferentially captured, which would affect the segregation ratio of alleles in the target areas. As to whether this has an impact on the prediction of parental haplotypes, further analysis is needed.

When inferring the parental haplotypes, the targeted hybrid capture panel provided more effective SNP sites, while when calculating the FF, it provided fewer SNP sites. Probably, the 120 SNP sites in the *HBB* gene region selected in the panel designed for multiple‐PCR amplification had a higher frequency of population distribution ratio and lower linkage, along with 300 SNP loci selected on the remaining chromosomes of the human genome. However, when designing the panel for probe hybridization capture, the region of the *HBB* gene was completely covered by the probes, showing a lack of tendency of the SNPs distribution in the target region.

When exploring the reasons for the failure of the prediction of the maternal haplotype inheritance, we found that the allelic separation ratios of heterozygous SNP of these pedigrees were significantly less than 0.5 in samples of mother, father, and proband, which may be affected by the enrichment method of the DNA. Probably when DNA was enriched by hybrid capture, the coverage of the probe was designed to be 1X in the targeted area when designing the panel, thus, it was possible to preferentially capture alleles with complementary sequences of the probes, which was also consistent with Figure 3. Whether such an allelic separation ratio has an impact on the prediction of the results requires further analysis. Moreover, the selected fetal concentration of 0.03 as the critical score requires further correction when DNA was enriched using multiple‐PCR amplification, due to its underestimation of the fetal concentration. However, in some cases, regardless of the fetal fraction, the allele separation ratio of each SNP, or the number of effective SNPs, the forecast result was still inaccurate. The reasons for the failure should be explored from the sample extraction quality, DNA library quality, and sequencing raw data quality analysis.

Positive prediction rates showed that the maternal haplotype prediction was not as accurate as paternal one, due to the large amount of maternal noise in the mother's peripheral blood. To distinguish the cffDNA from the maternal noise based on their biological characteristics, such as fragment length, breakpoints coordinates, GC content, more advanced bioinformatics methods are required.

This study selected one monogenetic disease to focus on, however, the haplotype‐based prenatal prediction method could also be applied to other types of single‐gene genetic diseases. The analysis method based on the core family requires collecting samples of the probands at the same time, which limits its clinical promotion and cannot be applied to families pregnant with their first child. Additionally, there were many ways to construct parental haplotypes. Recently, long‐read sequencing methods have been shown used as a more accurate and less complex parental haplotype analysis. HMM (Wang et al., 2017) and SPRT (Yang et al., 2020) are suitable for families with probands, and methods based on databases and populations are suitable for families without probands.

Currently, the NIPT of monogenetic diseases is still in the exploratory stage. The quality management and systems of the testing technologies need to be continuously standardized and improved before they can be clinically applied.

Current research needs to focus on the following aspects: First, the concentration of cfDNA fragments in the peripheral blood of pregnant women was low, and the DNA of the fetal origin was lower, which puts forward higher requirements for DNA enrichment and detection technology; and with the maternal DNA noise, the determination of the fetal genotype also required accurate biostatistical analysis methods. Second, in the SNP linkage analysis strategy, the distribution of SNPs was affected by factors such as population and race; the distribution of their allele frequency varied, so the number of effective SNP loci that could be detected in one family was limited, affecting the accuracy of the haplotype prediction. Third, the probability of recombination occurring was about 1% per 1 × 10^6^ bp in the human genome. With the lengthening of the sequenced fragments, the influence of the recombination on the results of fetal genotype inference needs to be verified. Finally, in both technologies adopted, it is necessary to shorten the time, simplify the steps, and reduce the cost while ensuring the accuracy of the haplotype construction.

## AUTHOR CONTRIBUTIONS

Du Hanxiao and Prof. Sun Luming contributed equally to this manuscript. Prof. Lu Daru and Prof. Jiang Ning designed the research; Prof. Sun Luming, Prof. Zhang Yueping, and Dr. Chen Songchang were responsible for samples collection; Dr. Yang Jingmin and Dr. Zhang Shuo performed the experiment, Du Hanxiao analyzed the data and wrote the manuscript; Prof. Jiang Ning and Prof. Chen Hongyan revised this thesis.

## FUNDING INFORMATION

Chongqing Natural Science Foundation cstc2020jcyj‐zdxm 0180 and cstc2019jxjl130001; National Natural Science Foundation of China (No. 81372706) and the National Key Research and Development Program of China (2021YFC2501800).

## CONFLICT OF INTEREST

The authors declare that they have no financial and personal relationships with other people or organizations that can inappropriately influence our work, there is no professional or other personal interest of any nature or kind in any product, service, and/or company that could be construed as influencing the manuscript entitled.

## ETHICS STATEMENT

This study was approved by the Ethics Committee of Taizhou Institute of Health Sciences, Fudan University (code: EC_AF_010).
